# Anti-Inflammatory Effects of Ginsenoside Rg3 via NF-*κ*B Pathway in A549 Cells and Human Asthmatic Lung Tissue

**DOI:** 10.1155/2016/7521601

**Published:** 2016-12-27

**Authors:** In-Seung Lee, InJoon Uh, Ki-Suk Kim, Kang-Hoon Kim, Jiyoung Park, Yumi Kim, Ji-Hoon Jung, Hee-Jae Jung, Hyeung-Jin Jang

**Affiliations:** ^1^College of Korean Medicine, Kyung Hee University, 26, Kyungheedae-ro, Dongdaemun-gu, Seoul 02447, Republic of Korea; ^2^Department of Science in Korean Medicine, Graduate School, Kyung Hee University, Seoul, Republic of Korea; ^3^Department of Biological Sciences in Korean Medicine, Graduate School, Kyung Hee University, Seoul, Republic of Korea

## Abstract

*Objective*. There is limited information of the anti-inflammatory effects of Rg3 on inflamed lung cells and tissues. Therefore, we confirmed the anti-inflammatory mechanism of ginsenoside Rg3 in inflamed human airway epithelial cells (A549) and tissues whether Rg3 regulates nuclear factor kappa B (NF-*κ*B) activity.* Methods*. To induce the inflammation, IL-1*β* (10 ng/ml) was treated to A549 cells for 4 h. The effects of Rg3 on NF-*κ*B activity and COX-2 expression were evaluated by western blotting analysis in both IL-1*β*-induced inflamed A549 cell and human asthmatic airway epithelial tissues. Using multiplex cytokines assay, the secretion levels of NF-*κ*B-mediated cytokines/chemokines were measured.* Result*. Rg3 showed the significant inhibition of NF-*κ*B activity thereby reduced COX-2 expression was determined in both IL-1*β*-induced inflamed A549 cell and human asthmatic airway epithelial tissues. In addition, among NF-*κ*B-mediated cytokines, the secretion levels of IL-4, TNF-*α*, and eotaxin were significantly decreased by Rg3 in asthma tissues. Even though there was no significant difference, IL-6, IL-9, and IL-13 secretion showed a lower tendency compared to saline-treated human asthmatic airway epithelial tissues.* Conclusion*. The results from this study demonstrate the potential of Rg3 as an anti-inflammatory agent through regulating NF-*κ*B activity and reducing the secretion of NF-*κ*B-mediated cytokines/chemokines.

## 1. Introduction

Asthma is an inflammatory disease which affects the airway of the lungs [1]. The repeated hysterical symptoms such as swell and narrow, leading to wheezing, limited breath, chest tightness, and coughing, are typical characters [2, 3]. It has become one of the most common health problems and a huge onus on healthcare costs worldwide [4]. The World Health Organization (WHO) has announced there was a sharp increase in the number of cases of asthma since the 1970s and the rates of increase show no signs of slowing. The exact cause of asthma has not been determined but studies have suggested that asthma resulted from a complex combination of genetic factors and environmental factors [5]. Ober and Hoffjan have been studying the genetic factors of asthma and they reported that over 100 genes were associated with asthma [6]. Many studies have proved there are two main factors of causing asthma: cause substance and deterioration factor. Cause substance, called allergen, is known to be dust mites, cockroaches, animal dander, and mold. The other type, deterioration factors, is air pollution, smoking, low air condition, climate change, stress, and yellow sand phenomenon [7, 8]. Allergic asthma, ordinarily related to atopy, is the most common type of asthma [9]. It is characterized by a reversible obstruction of the peripheral airway, induced by airway hyperresponsiveness (AHR), and infiltration of inflammation into the lung parenchyma [10, 11]. Atopy, the powerful risk factor of allergic asthma, is abnormally increased status of immunoglobulin E (IgE) which responds to allergen such as mold, animal, fur, and dust mites [12, 13]. Atopy is also involved in inflammation-related diseases such as allergic rhinitis, conjunctivitis, and atopic dermatitis [14].

The crucial role of type 2 helper cells (Th2 cells) immune pathway elements in AHR has been identified [15]. The effect of Th2 cells in inflammatory reaction of allergic asthma has been pointed out in several studies [16–18]. The antigen-presenting dendritic cells recognize allergen and active Th2 cells to stimulate the secretion level of cytokines including interleukin- (IL-) 4, 5, 9, and 13 and tumor necrosis factor-alpha (TNF-*α*) [11, 19–21]. IL-4 is known to lead Th2 cells differentiation. The function of IL-13 is similar to IL-4 [20]. Both IL-4 and IL-13 participate in alteration of IgE status in B cells [16, 17]. IL-5 causes the differentiation of eosinophils which leads to the release of proinflammatory mediators and reactive oxygen species (ROS) [22]. On the contrary, type 1 helper cells (Th1 cells) secrete interferon gamma (IFN-*γ*), IL-2, and IL-12, and those cytokines inhibit Th2 cells activation [23, 24]. Therefore, the main target of the treatment of allergic asthma is inhibiting Th2 cells-associated cytokines, IL-4, IL-5, and IL-13, or promoting Th1 cells-associated cytokines.

In response to innate and adaptive immune response regulating the release of cytokines through allergic asthma, nuclear factor kappa-light-chain-enhancer of activated B (NF-*κ*B) cells has been known to be involved in an activation of distinct signaling component through a cascade of phosphorylation [25]. NF-*κ*B, a protein complex, acts as a key role in regulating the immune response to infection [26, 27]. Because it regulates many genes involved in inflammation with several studies proved that NF-*κ*B is highly activated at sites of inflammation in various human diseases such as rheumatoid arthritis, atherosclerosis, multiple sclerosis, and asthma and also in the murine model [15, 28, 29]. When NF-*κ*B is activated, the combined form of p50 and p65 enters the nucleus and then p50 and p65 subunits bind with DNA thereby causing the expression of various gene products such as nitric oxide (NO), cyclooxygensase-2 (COX-2), and tumor necrosis factor-alpha (TNF-*α*) [30]. The former studies have proven that it is functionally related to the development of allergic disease of the airways by using mice that lacked p50 or c-Rel subunits of NF-*κ*B [31, 32].

Korean medicine has been interested in treatment modality among allergic disease patients [33]. Also, herbal medicines and its extracts have prescribed as an anti-inflammatory agent in clinical study [34]. Although herbal medicines have been known as the medicinal with antiallergic effects in clinical study, it was hard to find what component affected the anti-inflammation and regulated innate or adaptive immune response [33, 35]. Red ginseng (RG, derived from a steamed root of* Panax ginseng* Meyer) was chosen in this study, because the RG has been steadily used as a Korean medicine in clinical prescription and the anti-inflammatory effects in several studies have been researched [36–39]. Ginsenosides, so-called major component of Saponins, are the major component of RG [40]. Other studies have identified more than 40 ginsenosides in RG and most ginsenosides demonstrate various biological properties such as anti-inflammatory, antiallergic, and antitumor effects [41–43]. Various studies have reported that ginsenosides have an antiallergic effect on murine asthma and atopic disease models and among the ginsenosides Rh1 and Rh2 have antiallergic and anti-inflammatory effects [44, 45]. Ginsenoside Rg3 (Rg3) is the main component of RG [46]. Many studies have reported that Rg3 has an anti-inflammatory effect by reducing COX-2, inducible nitric oxide synthase (iNOS), and proinflammatory cytokines, including TNF-*α* and IL-1*β* expression, induced by LPS stimulation in vitro [47–49]. However, there is no study of the effects of Rg3 on human inflammation induced airway epithelial cells and human asthmatic airway epithelial tissue. Here, we examined how Rg3 affects the inflammatory reaction in IL-1*β*-induced inflamed human epithelial cells and human asthmatic airway epithelial tissue. And we demonstrated that Rg3 on activating condition of NF-*κ*B suppressed the proinflammatory cytokines secretion on human airway epithelial tissue.

## 2. Material and Methods

### 2.1. Materials

Rg3 and dexamethasone (Dex) were obtained from Sigma-Aldrich (St. Louis, MO, USA). Interleukin-1 beta (IL-1*β*) was obtained from R&D system Inc. (Minneapolis, MN, USA). Rabbit anti-phospho-NF-*κ*B p65, anti-NF-*κ*B p65, and anti-COX-2 were purchased from Cell Signaling Tech (Beverley, MA, USA). Mouse anti-*β*-actin, goat anti-rabbit IgG-HRP, and goat anti-mouse IgG-HRP were purchased from Santa Cruz Biotechnology (Dallas, TX, USA). Assay Media Sterile was purchased from MatTek Corp. (Ashland, MA, USA). Human Cytokine 6-plex Flat Bottom Express (TNF-*α*, eotaxin, IL-4, IL-5, IL-9, and IL-13) and protein assay reagent were purchased from Bio-Rad (Hercules, CA, USA). Protease inhibitor cocktail was obtained from Roche Diagnostics (Mannheim, Germany).

### 2.2. Cell Line and Culture Condition

A549 cells (American Type Culture Collection, Rockville, MD, USA), human airway epithelial cells, were used in all in vitro studies. The cells were cultured in RPMI 1640 medium (Corning Inc., NY, USA) containing 10% FBS (Gibco, Grand Island, NY, USA) and antibiotic and antimycotic (ABAM; Corning Inc., NY, USA) and incubated at 37°C and 5% CO_2_. Overnight serum starvation using Dulbecco's Modified Eagle Medium (DMEM: Corning Inc., NY, USA) containing low glucose without FBS was performed before IL-1*β*-induced inflammation at 37°C and 5% CO_2_.

### 2.3. Cell Viability Assay

MTT assays were performed to evaluate the cytotoxicity of Rg3 on inflamed cells. Ten thousands of A549 cells cultured each well of 96-well plate and were incubated at 37°C and 5% CO_2_ overnight. After serum starvation using DMEM low glucose without FBS, the medium was changed into RPMI containing IL-1*β* (10 ng/ml) and the cells were incubated at 37°C and 5% CO_2_ for 4 h. After 4 h incubation, the cells were treated with Rg3 (100–900 nM) for 12 h. Thirty microliters of MTT solution (5 mg/ml) was added to each well and the cells were incubated for 2 h. After 2 h incubation in cell culture incubator, the medium containing MTT solution of each well was removed and 50 *μ*l of dimethyl sulfoxide (DMSO; Sigma-Aldrich, MO, USA) was added. Using an automated spectrophotometric plate reader at 570 nm, the optical density of formazan was measured.

### 2.4. Human Asthmatic Airway Epithelial Tissue Culture

Asthmatic primary differentiated human airway epithelial tissues grown in 24-well plates on collagen coated transwell membrane inserts at an air-liquid interface were obtained commercially from MatTek Corporation (MatTek Corp., Ashland, MA, USA). Donors' information of asthmatic airway epithelial tissues is shown in Table 1. On arrival, the ex vivo epithelial tissues were washed with Dulbecco's Phosphate Buffered Saline (D-PBS) for removing collagen coating and carefully moved to 6-well plates that added 1 ml of Assay Media Sterile (AIR-100-ASY; MatTek Corp., Ashland, MA, USA) per each well. Tissues were equilibrated at 37°C and 5% CO_2_ for 16 h. After equilibration, the tissues were treated with saline, 1 *μ*M of Dex (Sigma-Aldrich, St. Louis, MO, USA), or 50 *μ*M of Rg3 for 24 h at 37°C and 5% CO_2_.

### 2.5. Western Blot Analysis

For the in vitro study, A549 cells were seeded in 6-well plate (5 × 10^5^ cells/well) and incubated at 37°C and 5% CO_2_ overnight. After serum starvation and inflammation induction using IL-1*β* (10 ng/ml), the cells were treated with Dex (5 *μ*M) or Rg3 (900 nM) for 12 h. After each drug treatment for 12 h, the cells were lysed by cell lysis buffer (Cell Signaling Tech., Beverley, MA, USA). The tissues were homogenized using the pestle. Each sample lysate from cell or tissue was centrifuged at 12,000 rpm for 20 min at 4°C. Supernatants were collected and transferred to fresh tube. Bio-Rad protein assay reagent (Bio-Rad, Hercules, CA, USA) was used for measuring protein concentrations. Each sample was separated on a 10% SDS-PAGE gel at 120 V for 120 min and then electrotransferred to a NC membrane for 100 min at 25 V. The membranes were blocked with 5% BSA in TBST (Tris-buffered saline with 0.1% Tween 20 and 0.04% NaN_3_). Primary antibodies, that is, rabbit anti-phospho-NF-*κ*B p65, rabbit anti-NF-*κ*B p65, rabbit anti-COX-2, and mouse anti-*β*-actin, were stained onto the membrane in 1 : 1,000 dilutions (anti-*β*-actin was 1 : 3,000 dilution) for overnight. Membranes were extensively washed and then incubated with the following secondary antibodies: goat anti-rabbit IgG-HRP and goat anti-mouse IgG-HRP. Bands were visualized with an enhanced chemiluminescence (ECL) kit (EMD Millipore Co., Billerica, MA, USA) and were quantified with the Image J program (National Institutes of Health, Bethesda, MD, USA).

### 2.6. Analysis of Proinflammatory Cytokines in Human Asthmatic Airway Epithelial Tissues

After indicated drugs treatment for 24 h on human asthmatic airway epithelial tissues, each medium was collected and centrifuged at 1,000 ×g for 10 min. The centrifuged supernatant was transferred to fresh tubes. Human Cytokine 6-plex Flat Bottom Express (TNF-*α*, eotaxin, IL-4, IL-5, IL-9, and IL-13; Bio-Rad, Hercules, CA, USA) assay was performed according to the manufacturer's guidelines. Briefly, 50 *μ*l of centrifuged samples was transferred to each well that included human cytokine 6-plex magnetic beads: IL-4, IL-5, IL-9, IL-13, TNF-*α*, and eotaxin. The plate was incubated on shaker at 850 rpm for 30 min at room temperature (RT). After 30 min of shaking incubation, the solution of each well was removed and changed into 25 *μ*l of detection antibody mixed solution and incubated on shaker at 850 rpm for 30 min at RT. During 30 min incubation, streptavidin-PE (SA-PE) was diluted to assay buffer (Cat. number 10014822, Bio-Rad, Hercules, CA, USA), and then 50 *μ*l of SA-PE diluted solution was shifted to wells. Finally, 125 *μ*l of assay buffer was added to each well and measured by a Bio-Plex® MAGPIX™ Multiplex reader (Bio-Rad, Hercules, CA, USA).

### 2.7. Statistical Analysis

Data were represented with the means ± SEM. The GraphPad Prism 5 software package (GraphPad Software, San Diego, CA, USA) was used to conduct the statistical analysis of the results. Unpaired *t* test (one-tailed) was conducted to analyze western blot band and multiplex assay results. *P* value less than 0.05 was considered to be statistically significant.

## 3. Results

### 3.1. Effects of Rg3 on Cell Viability

**Figure 1:**
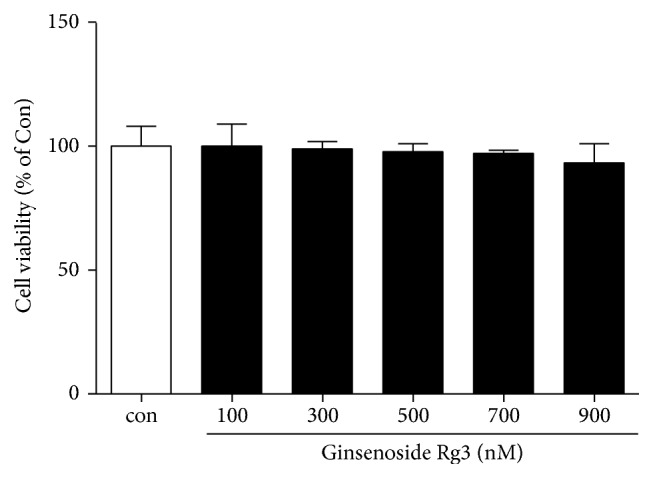
Cell viability assay of Rg3 on IL-1*β*-induced inflamed A549 cells. After treatment of IL-1*β* (10 ng/ml) and Rg3 (100–900 nM) on the A549 cells, MTT assay was performed. The cytotoxicity of Rg3 on IL-1*β*-induced inflamed A549 cells was not detected. Statistical significance was determined by unpaired *t* test (one-tailed) compared to Con (only treated PBS).

To examine the cytotoxicity of Rg3 on IL-1*β*-induced inflamed A549 cells, the cells were firstly treated with IL-1*β* (10 ng/ml) for 4 h and treated with 100 to 900 ng/ml concentration of Rg3 for 12 h. Cell viability was analyzed using an MTT assay. There was no observed cytotoxicity of Rg3 in IL-1*β*-induced inflamed A549 cells compared to only PBS-treated cells (Con) (Figure 1).

### 3.2. Inhibition of IL-1*β*-Stimulated NF-*κ*B Expression by Rg3 in Inflamed A549 Cells

**Figure 2:**
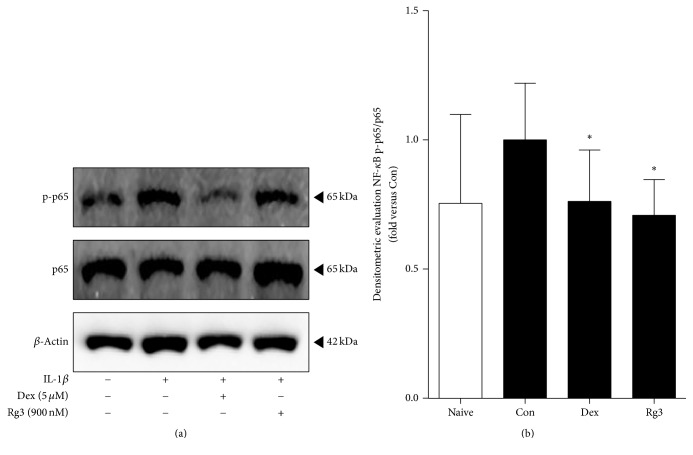
Effects of Rg3 on NF-*κ*B pathway in IL-1*β*-induced inflamed A549 cells. (a) The western blot results in representative three separate experiments. (b) Ratio of phospho-p65/p65 expression was measured by Image J program. Statistical significance was determined by unpaired *t*-test (one-tail) compared to Con. Values are means ± SEM. ^*∗*^*P*< 0.05. Naive: normal condition; Con: IL-1*β* treated A549 cells.

To obtain the anti-inflammatory effects of Rg3 on inflammation induced human lung epithelial cells, A549 cells inflammation was induced by IL-1*β* (10 ng/ml) and then treated by 5 *μ*M of Dex or 900 nM of Rg3. The NF-*κ*B activation was analyzed by a western blot analysis to evaluate the effect of Rg3 treatment on A549 cells (Figure 2(a)). The NF-*κ*B pathway plays a central role in immune and inflammatory responses, because the nuclear translocation of the NF-*κ*B p65 subunit stimulates the transcription of various proinflammatory genes. Phospho-NF-*κ*B p65/total NF-*κ*B p65 densitometry in the cells treated with Rg3 showed the significant decrease compared to IL-1*β*-induced inflamed A549 cells (Figure 2(b)). The meaning of reducing the ratio of p-p65/p65 by Rg3 treatment is associated with NF-*κ*B activation; thereby inflammation may be reduced.

### 3.3. Suppression Effects of Rg3 on COX-2 Protein Expression in IL-1*β*-Induced Inflamed A549 Cells

**Figure 3:**
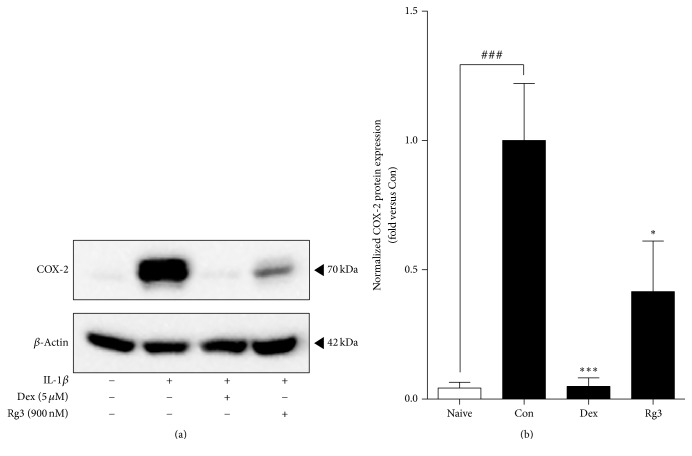
Inhibited IL-1*β*-stimulated COX-2 expression by Rg3 in inflamed A549 cells. (a) The western blot results in representative three separate experiments. (b) COX-2 expression level normalized with *β*-actin was measured by densitometry analysis and represented as bar charts. Statistical significance was determined by unpaired *t* test (one-tail) compared to Con. Values are means ± SEM. ^###^*P*< 0.001; ^*∗*^*P*< 0.05; ^*∗∗∗*^*P*< 0.001. Naive: normal condition; Con: IL-1*β* treated A549 cells.

As we examined whether Rg3 suppresses expression of a downstream mediator of NF-*κ*B activation, we investigated COX-2 expression, known to be a downstream mediator of NF-*κ*B activation. COX-2 is the enzyme that makes prostaglandins which arouse inflammation, pain, and fever. Thereby, we also investigated the expression of COX-2 in IL-1*β*-induced inflamed A549 cells. We found that Rg3 downregulated the expression of COX-2 effectively (Figure 3).

### 3.4. Effects of Rg3 on NF-*κ*B Activation in Human Asthmatic Airway Epithelial Tissues

**Figure 4:**
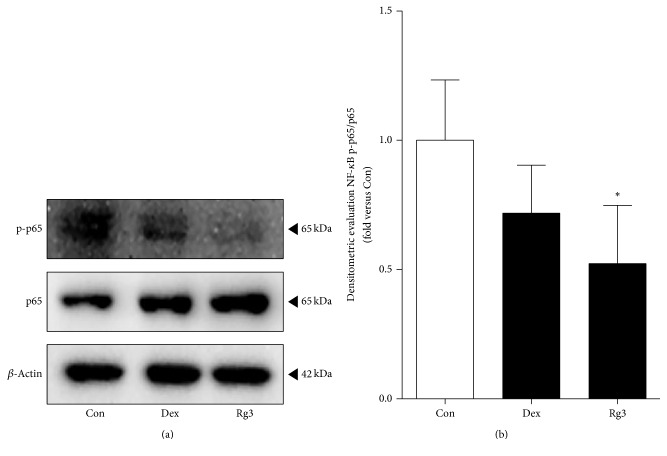
Suppressed NF-*κ*B activation by Rg3 in human asthmatic airway epithelial tissues. (a) Human asthmatic lung tissue was treated with Dex for positive control or Rg3, and whole tissue lysates were prepared and then subjected to western blot analysis with anti-NF-*κ*B phospho-p65 or anti-NF-*κ*B p65 antibodies. Anti-*β*-actin antibody was used as an internal control. (b) Ratio of phospho-p65/p65 expression was measured by Image J program and represented as bar charts. Statistical significance was determined by unpaired *t* test (one-tail) compared to Con (only PBS-treated asthmatic lung tissue). Values are means ± SEM; ^*∗*^*P*< 0.05.

From the inhibitory effect of Rg3 on NF-*κ*B activity in the inflamed airway epithelial cells, we anticipated that Rg3 may also inhibit NF-*κ*B activation in human asthmatic airway epithelial tissues. The NF-*κ*B activation was analyzed by a western blot analysis to evaluate the effect of Rg3 treatment on the tissues (Figure 4(a)). Human asthmatic airway epithelial tissue was treated with saline, 1 *μ*M of Dex, and 50 *μ*M of Rg3. We observed that there was a trend toward decreased phospho-NF-*κ*B p65/total NF-*κ*B p65 densitometry in the tissue treated with Rg3 (Figure 4(b)). The treatment of Rg3 on human asthmatic airway epithelial tissue showed a significant decrease in densitometry of p-p65/p65; thereby inflammation may be reduced.

### 3.5. Rg3 Suppresses IL-1*β*-Induced COX-2 Expression in Human Asthmatic Airway Epithelial Tissues

**Figure 5:**
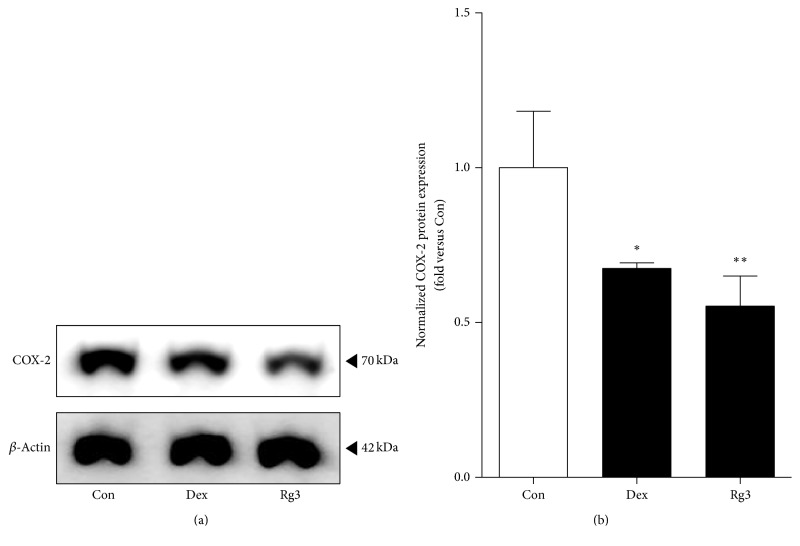
Effect of Rg3 on COX-2 protein inhibition in human asthmatic airway epithelial tissues. (a) Human asthmatic lung tissue was treated with Dex for positive control or Rg3, and whole tissue lysates were prepared and then subjected to western blot analysis with anti-COX-2 antibodies. Anti-*β*-actin antibody was used as an internal control. The blots were normalized by *β*-actin expression levels and compared to the saline-treated tissue. (b) Normalized COX-2 expression was measured by Image J program and represented as bar charts. Statistical significance was determined by unpaired *t* test (one-tail) compared to Con (only PBS-treated asthmatic lung tissue). Values are means ± SEM; ^*∗*^*P*< 0.05; ^*∗∗*^*P*< 0.01.

As we found the reducing effects of Rg3 on COX-2 expression in inflamed cells, we investigated the effects of Rg3 on the protein expression level of COX-2 in human asthmatic airway epithelial tissues. The protein expression level of COX-2 was also analyzed by a western blot analysis (Figure 5(a)). We found the significant inhibitory effects of Rg3 on COX-2 expression (Figure 5(b)).

### 3.6. Inhibition of NF-*κ*B-Mediated Proinflammatory Cytokines Secretion by Rg3 in Human Asthmatic Airway Epithelial Tissues

**Figure 6:**
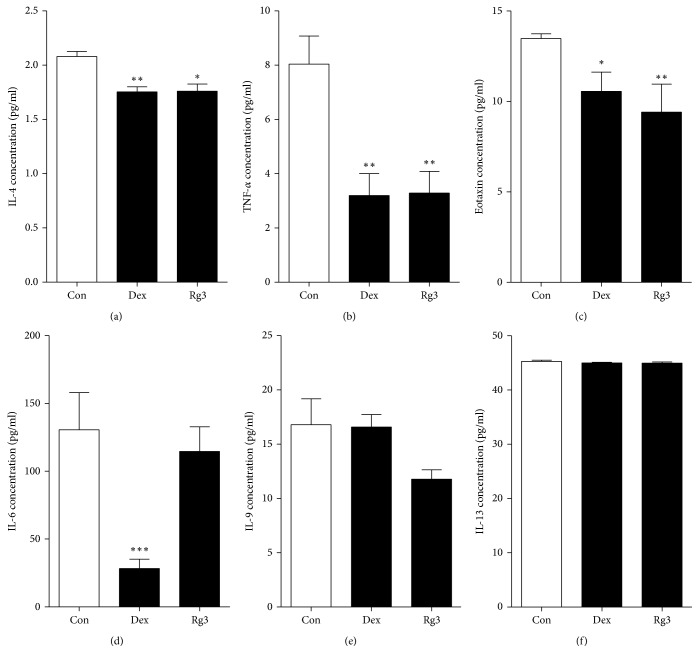
Anti-inflammatory effect of Rg3 on NF-*κ*B-mediated proinflammatory cytokine/chemokine levels in human asthmatic airway epithelial tissues. Rg3 suppresses NF-*κ*B-mediated proinflammatory cytokines IL-4, TNF-*α*, and eotaxin secretion (a–c). IL-6, IL-9, and IL-13 had no statistical significance (d–f). Statistical significance was determined by unpaired *t* test (one-tailed) compared to Con (only PBS-treated asthmatic lung tissue), and the values are means ± SEM; ^*∗*^*P*< 0.05; ^*∗∗*^*P*< 0.01, ^*∗∗∗*^*P* < 0.001.

The cytokines/chemokines levels in media were determined by a multiplex assay, as described in Material and Methods. Specifically, IL-4, TNF-*α*, and eotaxin concentration levels were significantly decreased in the tissues treated with Rg3 compared to saline-treated tissues (Figures 6(a)–6(c)). Although there is no significant difference, the decreased tendencies of IL-6, IL-9, and IL-13 secretion levels were shown in Rg3-treated human asthmatic airway epithelial tissue compared to saline-treated tissue (Figures 6(d)–6(f)). The data of that cytokine/chemokine associated with Th2-cells support our hypothesis that Rg3 may have an anti-inflammatory effect by inhibiting NF-*κ*B activation.

## 4. Discussion

The study was to determine whether Rg3 exerts anti-inflammatory effects through the attenuation of the NF-*κ*B signaling pathways in inflamed human airway epithelial cell and human asthmatic airway epithelial tissues and thereby whether Rg3 affects NF-*κ*B-mediated proinflammatory cytokines in human asthmatic airway epithelial tissues. Once considered to largely act as a barrier function in the lung, airway epithelium has been widely recognized for its immunomodulatory capabilities. Finally, there was the evaluation of the suppression of IL-1*β*-induced inflammation by Rg3 in human airway epithelial cell. We conclusively demonstrated that Rg3 inhibited IL-1*β*-induced NF-*κ*B activation in human airway epithelial cell and also Rg3 suppressed NF-*κ*B activity in human asthmatic airway epithelial tissues and, as a result, Rg3 downregulated the expression of NF-*κ*B-mediated gene products such as COX-2, IL-4, TNF-*α*, and eotaxin.

NF-*κ*B, a protein complex, acts as a major player in the pathogenesis of inflammation regulating the expressions of multiple genes including iNOS, COX-2, and cytokines [25, 26]. A study has reported that upregulated NF-*κ*B activity relates to asthmatic inflammation in human and animal lung tissues [50]. Thus, we evaluated the suppression effect of Rg3 on IL-1*β*-stimulated NF-*κ*B activity in human airway epithelial cell (Figure 2). The next step was whether Rg3 also blocked the NF-*κ*B activity and we investigated proinflammatory cytokines secretion in human asthmatic airway epithelial tissues using western blotting analysis and multiplex assay, respectively (Figures 4 and 6).

We confirmed the reduction of NF-*κ*B expression by Rg3 in both inflamed A549 cells and human asthmatic airway epithelial tissues (Figures 2 and 4). We also confirmed that Rg3 inhibited the COX-2 expression, known as the downstream of NF-*κ*B activity. COX-2 is an enzyme that makes prostaglandins E_2_ (PGE_2_), which leads to inflammation and pain [51]. COX-2, identified in epithelial cells, macrophages, fibroblasts, smooth muscle cells, and mast cells, has important roles in immunity, renal physiology, neurotransmission, bone reabsorption, and pancreatic secretion [52, 53]. At first we found out the upregulated expression level of COX-2 by 10 ng/ml of IL-1*β* treatment in human airway epithelial cell. And the high expression level of COX-2 appeared in human asthmatic airway epithelial tissues. We confirmed that IL-1*β*-stimulated COX-2 expression level was significantly reduced by Rg3 in human airway epithelial cell (Figure 3) and in human asthmatic airway epithelial tissues was also confirmed (Figure 5). As mentioned above, the reduction of COX-2 expression by Rg3 through inhibiting NF-*κ*B activity leads to inhibition of the production of PGE_2_.

Many studies reported that cytokines and chemokines are involved in the pathophysiology of asthma [9, 19]. The presence of IL-4 induces the naïve T cell to Th2 cell and then the differentiated Th2 cells regulated cytokines. Among these cytokines, IL-1, IL-6, and TNF-*α* are considered to be involved in various inflammatory diseases including asthma and chronic pulmonary disease (COPD) [54]. As aforementioned, IL-4 encourages the naïve T cell to differentiate Th2 cell; thereby it regulates allergic inflammation. In addition, IL-4 induces IgE synthesis in the pathogenesis of asthma. IL-5 induces eosinophilic inflammation. IL-9 stimulates cell proliferation and regulates apoptosis. IL-13 has similar role as IL-4 including IgE synthesis [16, 17]. When IL-4 and IL-13 are highly expressed, this state leads mucus hypersecretion and eosinophil infiltration to the airway tissues [55]. TNF-*α* is involved in systemic inflammation and plays an important role in endotoxemia. Increased TNF-*α* production levels improve the procoagulant activity of vascular endothelial cells, initiate macrophages, and increase adherent molecule expression, thus it enhances neutrophil infiltration [56]. In addition, TNF-*α* induces the PGE_2_ production via activating COX-2. Eotaxin is a chemokine subfamily of eosinophil chemotactic protein. It is involved in immune reaction in human and animal body [10]. In this study, we observed that Rg3 significantly reduced the secretion of IL-4, TNF-*α*, and eotaxin in human asthmatic airway epithelial tissues through inhibiting NF-*κ*B activity. Even though the significant reduction was not determined, there were trends toward decrease in the secretion of IL-6, IL-9, and IL-13 (Figure 6). The results from reduced cytokines secretion by Rg3 demonstrate that Rg3 may regulate the synthesis of IgE and the differentiation of naïve T cell to T_h_2 cell through blocking IL-4 secretion and the eosinophil responses via inhibitory effect on eotaxin secretion. In addition, Rg3 also may suppress the PGE_2_ production via blocking TNF-*α* secretion; thereby inflammation, fever, and pain followed by PGE_2_ production may decrease.

Due to the minimized side effects and excellent anti-inflammatory effects of traditional Korean medicines, they are good options for treatment of asthmatic airway disease. Red ginseng has been widely applied as a Korean medicine in clinical prescription and has been used traditionally in oriental countries to improve health [57, 58]. Ginsenosides are the major components of RG and more than 40 ginsenosides in RG were identified [40]. Rg3 is one of major components of RG [46]. Many studies have been reporting that Rg3 exhibits in vitro and in vivo anticarcinogenic and antimetastatic effects and also Rg3 exerted inhibitory effect on proliferation, capillary tube formation, and invasion of human umbilical vein endothelial cells (HUVEC) [59–61]. In addition, Rg3 has anti-inflammatory effects by inhibiting COX-2, iNOS expression, and proinflammatory cytokines production in LPS-induced cells [47, 48].

## 5. Conclusion

In conclusion, our data suggested that Rg3 regulates the inflammation reaction in the airways via inhibiting NF-*κ*B activity, an important role in inflammation due to its stimulus effect on the transcription of various proinflammatory gene, in IL-1*β*-induced inflamed human airway epithelial cell and human asthmatic airway epithelial tissues. Thus COX-2 expression and the secretion NF-*κ*B-mediated proinflammatory cytokines including IL-4, TNF-*α*, and eotaxin were significantly reduced in human asthmatic airway epithelial tissues. Even though there was no significant reduction, the decrease tendency was demonstrated in the secretion of IL- 6, IL-9, and IL-13. This study demonstrates the inhibitory mechanism of ginsenoside Rg3 through the attenuation of NF-*κ*B activity in inflammation induced human airway epithelial cell and human asthmatic airway epithelial tissues. Thereby, Rg3 may be qualified as a potential remedial agent for asthma by inhibiting NF-*κ*B activation.

## Figures and Tables

**Table 1:** Description of asthmatic human airway epithelial tissue donors.

Age	Sex	Race	Smoking	Disease	Cause of death	Medication
7	F	C	N	Asthma		Albuterol
9	F	B	N	Asthma since birth	Possible asthma attack	Albuterol
16	M	B	N	Asthma		Advair

M, male; F, female; C, Caucasian; B, black; N, none.
